# Increased co-expression of stromal HHLA2 and fibroblast activation protein in upper tract urothelial carcinoma

**DOI:** 10.1007/s11255-022-03458-0

**Published:** 2023-01-04

**Authors:** Daisaku Nishihara, Toshiki Kijima, Kyoko Arai, Takao Kamai

**Affiliations:** Department of Urology, Dokkyo Medical University, 880 Kitakobayashi, Mibu, Shimotsuga, Tochigi 321-0293 Japan

**Keywords:** HHLA2, B7-H7, Fibroblast activation protein, Cancer-associated fibroblast, Upper tract urothelial carcinoma

## Abstract

**Background:**

The human endogenous retrovirus-H long terminal repeat-associating protein 2 (HHLA2; also known as B7 homolog 7 [B7-H7]) regulates immune responses. However, its immunoregulatory role in upper tract urothelial carcinoma (UTUC) remains unclear.

**Methods:**

We evaluated the immunohistochemical expression of HHLA2 and fibroblast activation protein (FAP), which is a marker of cancer-associated fibroblasts, in UTUC tissues from 85 patients who underwent nephroureterectomy. The associations between the expressions of HHLA2 and FAP and clinicopathological characteristics were investigated.

**Results:**

The increased expression of HHLA2 in tumor cells (t-HHLA2) was associated with a low histological grade, a negative lymphovascular invasion (LVI), and a low neutrophil-to-lymphocyte ratio, whereas an increased expression of HHLA2 in stromal cells (s-HHLA2) was associated with a high histological grade. No correlation was observed between the expression of t-HHLA2 and s-HHLA2. FAP was expressed only in the stromal cells (s-FAP). Positive s-FAP expression was significantly associated with increased s-HHLA2 expression, higher histological grade, higher pathological T stage, and positive LVI. Higher t-HHLA2 was associated with longer cancer-specific and progression-free survival. In contrast, positive s-FAP was associated with short progression-free survival.

**Conclusion:**

These findings suggest that the progression of UTUC may involve increased co-expression of HHLA2 and FAP in the tumor stroma.

## Introduction

The prevalence of upper tract urothelial carcinoma (UTUC) is gradually increasing [1]. Radical nephroureterectomy is the standard treatment for localized UTUC; however, the prognosis is poor because of frequent recurrence and metastases. With the recent success of cancer immunotherapy targeting immune checkpoints such as programmed cell death receptor 1 (PD-1) and programmed cell death 1 ligand 1 (PD-L1; also known as B7 homologs 1 [B7-H1]) [2, 3], anti-PD-1 antibody pembrolizumab is currently being investigated as a potential neoadjuvant therapy for UTUC [4]. However, the response rates to PD-1/PD-L1 inhibitors in urothelial carcinoma are unsatisfactory, indicating the presence of additional immune-inhibitory pathways other than the PD-1/PD-L1 pathway that contribute to tumor immune evasion.

Tumor cells evade the immune system by hijacking immune cells in the tumor microenvironment (TME) [5]. In the TME, tumor, stromal, and immune cells upregulate the inhibitory B7 family molecules, which inhibit T cell activation and ultimately protect tumor cells from the immune response [6]. The human endogenous retrovirus-H long terminal repeat-associating protein 2 (HHLA2; also known as B7-H7) is the most recently discovered ligand of the B7 family. HHLA2 mediates co-stimulatory signals by interacting with the transmembrane and immunoglobulin domain-containing 2 (TMIGD2), a member of the CD28 family, and also mediates inhibitory signals via undetermined receptors on T cells [7, 8]. HHLA2 inhibits T cell activation and proliferation in the presence of co-stimulatory CD28 signaling via TMIGD2, similar to the co-inhibitory functions of PD-L1 and B7-H4 [9]. Bhatt et al. recently identified killer cell immunoglobulin-like receptors, three immunoglobulin domains, and long cytoplasmic tail 3 (KIR3DL3) as an inhibitory receptor of HHLA2 expressed on T cells and natural killer cells [10]. Although HHLA2 has been established as a novel immune checkpoint molecule, its role in tumor progression and tumor immune evasion remains unclear.

Previous studies have focused on the interaction between tumor and stromal cells in TME as a mechanism of cancer progression and a potential therapeutic target. Cancer-associated fibroblasts (CAFs) are activated fibroblasts that produce multiple tumor components. CAFs may induce tumor progression by inhibiting anti-tumor immunity. For example, CAFs affect the localization of T cells, and the presence of CAFs is associated with resistance to checkpoint inhibitors [11]. CAFs are characterized by the expression of α-smooth muscle actin, fibronectin, vimentin, and fibroblast activation protein (FAP) which is a cell-surface serine protease that acts on extracellular matrix components. FAP, which has been utilized as a marker for CAF, is highly upregulated in various cancers and is shown to be a poor prognostic factor [12].

In this study, we aimed to investigate the prognostic significance of HHLA2 in surgically treated non-metastatic UTUC patients. We also evaluated the possible interaction between HHLA2 expression in tumor cells and stromal cells, and CAFs in TME. As the prognostic impact of the expression of HHLA2 and the interaction between HHLA2 and CAFs have not been reported in human UTUC, this study will provide novel insights regarding the role of immune checkpoints and CAFs in the progression of UTUC.

## Patients and methods

### Patients

This retrospective study included 85 patients (67 male, 18 female; median age: 70 y; age range: 42–85 y) who underwent nephroureterectomy at Dokkyo Medical University Hospital from 2010 to 2020. The follow-up data were updated until the end of September 2021. All patients had UTUC either of the renal pelvis (*n* = 40) or the ureter (*n* = 45), with clinical stage TanyN0M0. The clinical stage of the UTUC was determined via computed tomography (CT) and/or magnetic resonance imaging scan performed before surgical resection, according to the 2017 TNM classification of malignant tumors [13]. The patients did not receive any neoadjuvant/adjuvant chemotherapy. Lymph node dissection was not performed routinely but only when nodal involvement was suspected during surgery. During follow-up, patients underwent CT scans every 3 mo for the first 2 y and every 6 mo thereafter. Cancer progression was defined as the development of local recurrence or metastases. During the median postoperative follow-up period of 34 mo (range: 3–123 mo), 40 (47%) experienced a recurrence and 25 (29%) died of cancer. After the confirmation of recurrence, 31 (36%) of the 85 patients received platinum-based chemotherapy, and 8 (9%) received anti-PD1 pembrolizumab therapy.

The white blood cell, neutrophil, and lymphocyte counts and the neutrophil-to-lymphocyte ratio (NLR) before surgery were obtained from the medical records of the patients. An NLR cut-off of 3.5 was used to divide patients into two groups: the lower NLR group with NLR ˂ 3.5 and the higher NLR group with NLR ≥ 3.5.

This study followed the ethical guidelines set by the Declaration of Helsinki. The study protocol was approved by the Dokkyo Medical University Hospital ethics committee (R-31-10 J). All patients provided written informed consent via a consent form approved by the hospital’s Committee on Human Rights in Research for their data to be included in the study.

### Immunohistochemistry

Surgically resected tumor tissue specimens were sectioned at a thickness of 4 μm, fixed in formalin, and embedded in paraffin. Immunohistochemical analysis was performed using the rabbit polyclonal anti-HHLA2 antibody (Abcam, ab207178, Cambridge, UK) and the rabbit monoclonal anti-FAP antibody (Abcam, ab207178, Cambridge, UK). Immunohistochemical staining was performed using the automated BOND-III system (Leica Biosystems Newcastle Ltd., Newcastle, UK), according to the manufacturer’s instructions [14]. Healthy tissues from the colon and the placenta were used as positive controls for HHLA2 expression.

To quantify the expression levels of HHLA2 and FAP, the proportion of the area positive for HHLA2 and FAP was calculated by computer-assisted cytometrical analysis with the WinROOF image processing software (Mitani Corp., Tokyo, Japan) as reported previously [15]. Expression of HHLA2 was observed either in tumor cells or in stromal cells including fibroblasts and mesenchymal stromal cells. A cut-off value of 20% for HHLA2 in tumor cells (t-HHLA2) was determined by the receiver-operating characteristic (ROC) curve and was applied to divide the patients into two groups: higher t-HHLA2 (area positive for t-HHLA2 ≥ 20% of the total tumor area) and lower t-HHLA2 (area positive for t-HHLA2 < 20% of the total tumor area). Similarly, we used an ROC curve to determine the cut-off for HHLA2 in the stromal cells (s-HHLA2) and divided the patients into two groups: higher s-HHLA2 (area positive for s-HHLA2 ≥ 5% of the stromal area) and lower s-HHLA2 (area positive for s-HHLA2 < 5% of the stromal area).

FAP expression was detected only in stromal cells and not in tumor cells. As FAP was assumed as the marker of CAFs, we expect all stromal cells positive for FAP were CAFs. The patients were divided into two groups: positive FAP expression (area positive for FAP ≥ 1% of the stromal area) and negative FAP expression (area positive for FAP < 1% of the stromal area).

### Statistical analysis

The association between the expressions of HHLA2 and FAP and clinicopathological findings were analyzed using Fisher's exact test. Progression-free survival (PFS) and cancer-specific survival (CSS) were estimated using the Kaplan–Meier method, and the differences in survival were assessed using the log-rank test. The impact of various factors on survival was assessed through Cox’s proportional hazards analysis. For all analyses, *p* ˂ 0.05 was considered to be statistically significant. The analyses were performed using JMP 13.0 statistical software (SAS Institute, Cary, NC, USA).

## Results

**Fig. 1:**
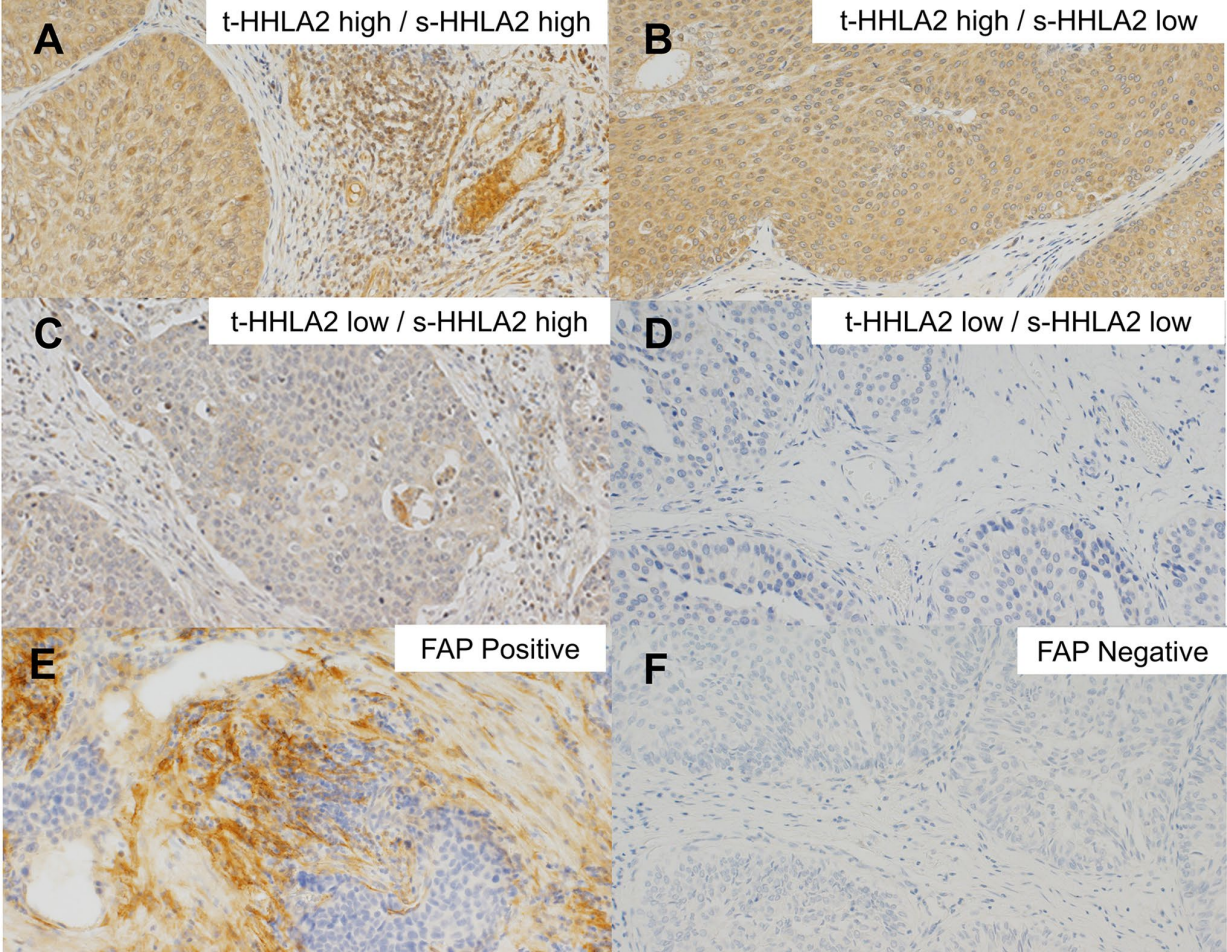
Representative images of the immunohistochemical analyses of HHLA2 and FAP. **a** Tumor HHLA2 high – stromal HHLA2 high, **b** Tumor HHLA2 high – stromal HHLA2 low, **c** Tumor HHLA2 low – stromal HHLA2 high, **d** Tumor HHLA2 low – stromal HHLA2 low, **e** FAP positive, and **f** FAP negative

**Fig. 2:**
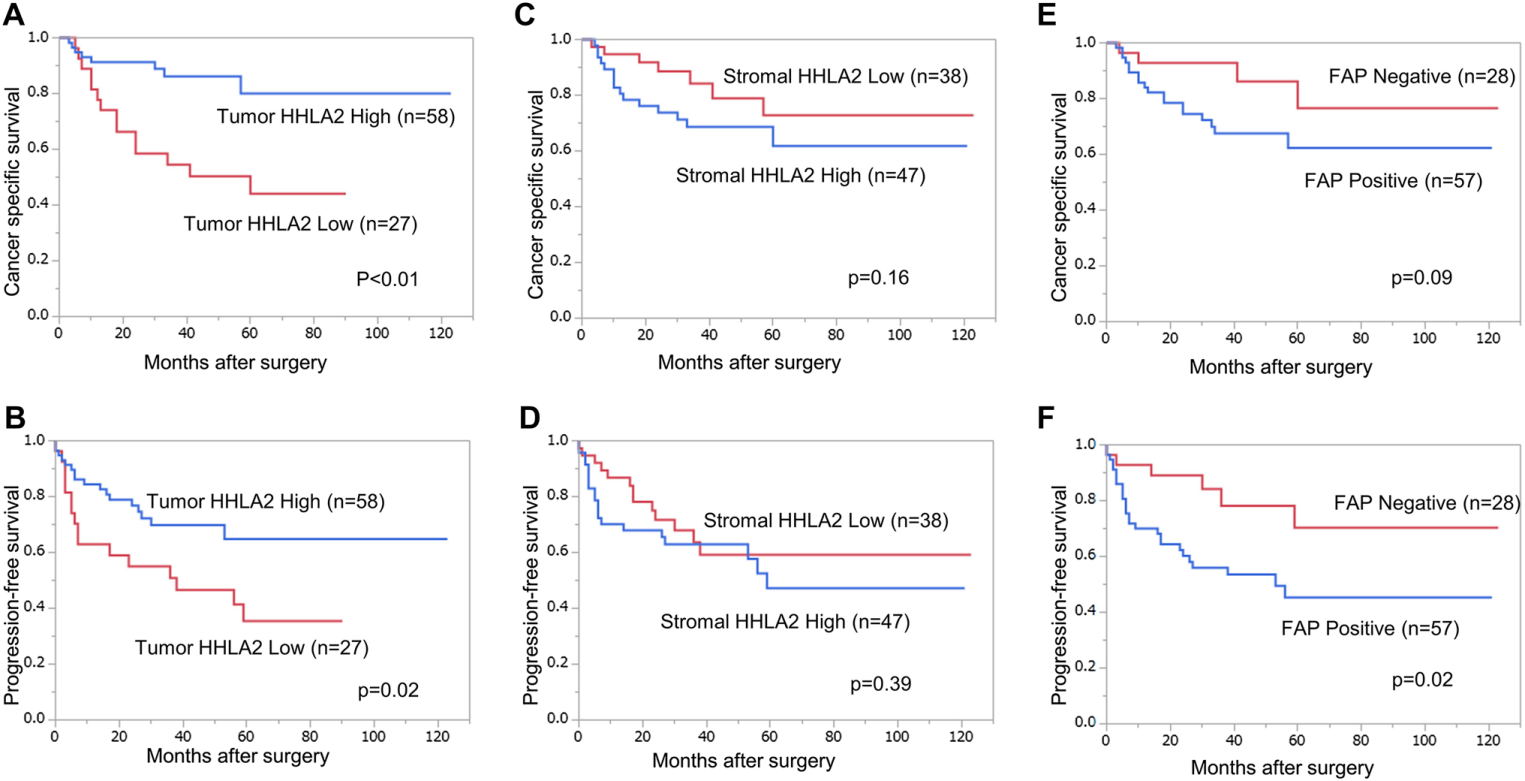
Cancer-specific survival (CSS) and progression-free survival (PFS) of the patients. CSS and PFS according to **a** Tumor HHLA2 expression, **b** Stromal HHLA2 expression, and **c** Stromal FAP expression. PFS according to **d** Tumor HHLA2 expression, **e** Stromal HHLA2 expression, and **f** Stromal FAP expression

The expression of t-HHLA2 was detected both on the membrane and in the cytoplasm of the tumor cells (Fig. 1A–D). The increased expression of t-HHLA2 was associated with a lower histological grade, negative lymphovascular invasion (LVI), and lower NLR. In contrast, increased expression of s-HHLA2 was associated with a higher histological grade (Table 1). The Kaplan–Meier estimates showed that an increased t-HHLA2 expression was related to longer CSS (*p* < 0.01, Fig. 2A) and PFS (*p* = 0.02, Fig. 2B). In contrast, an increased s-HHLA2 expression was not associated with CSS or PFS (Fig. 2C, D). No correlation was observed between the expressions of t-HHLA2 and s-HHLA2 (*p* = 0.65). These findings suggest that t-HHLA2 and s-HHLA2 may have different effects on the TME and clinical outcomes in UTUC patients. Hence, we further analyzed the tumor stromal cells using the anti-FAP antibody.

**Table 1:** Clinicopathological characteristics of the patients stratified by HHLA2 and FAP expression

		Patients		Tumor HHLA2 expression			Stromal HHLA2 expression			Stromal FAP expression		
Clinicopathological characteristics		*N*	%	Low	High	*P* value	Low	High	*P* value	Negative	Positive	*P* value
Age	≦ 70	43	51	12	31	0.44	15	28	0.08	14	29	1.00
	> 70	42	49	15	27		23	19		14	28	
Gender	Male	67	79	18	49	0.06	33	34	0.12	26	41	0.04*
	Female	18	21	9	9		5	13		2	16	
Pathological T stage	≦ T2	48	57	12	36	0.12	22	26	0.83	23	25	< 0.01*
	≧ T3	37	43	15	22		16	21		5	32	
Tumor location	Renal pelvis	40	47	12	28	0.74	20	20	0.39	16	24	0.25
	Ureter	45	53	15	30		18	27		12	33	
Histological type	Pure UC	76	89	26	50	0.16	36	40	0.18	25	51	1.00
	UC variants	9	11	1	8		2	7		3	6	
Grade	1, 2	51	60	12	39	0.04*	28	23	0.03*	22	29	0.02*
	3	34	40	15	19		10	24		6	28	
LVI	Negative	51	60	9	42	< 0.01*	26	25	0.19	23	28	< 0.01*
	Positive	34	40	18	16		12	22		5	29	
Serum CRP levels	≦ 0.5	67	80	21	46	0.76	29	38	0.80	26	41	0.04*
	> 0.5	17	20	6	11		8	9		2	15	
Serum Alb levels	< 3.5	10	12	5	5	0.19	4	6	1.00	3	7	1.00
	≧3.5	75	88	22	53		34	41		25	50	
NLR	≦3.5	73	86	19	54	0.02*	33	40	1.00	25	48	0.74
	> 3.5	12	14	8	4		5	7		3	9	
TIL in stromal cells	≦ 5%	12	14	4	8	1.00	6	6	0.76	5	7	0.52
	> 5%	73	86	23	50		32	41		23	50	

*Alb* albumin, *CRP* c-reactive protein, *LVI* lymphovascular invasion, *NLR* neutrophil lymphocyte ratio, *TIL* tumor-infiltrating lymphocyte, *UC* Urothelial carcinoma

**Fig. 3:**
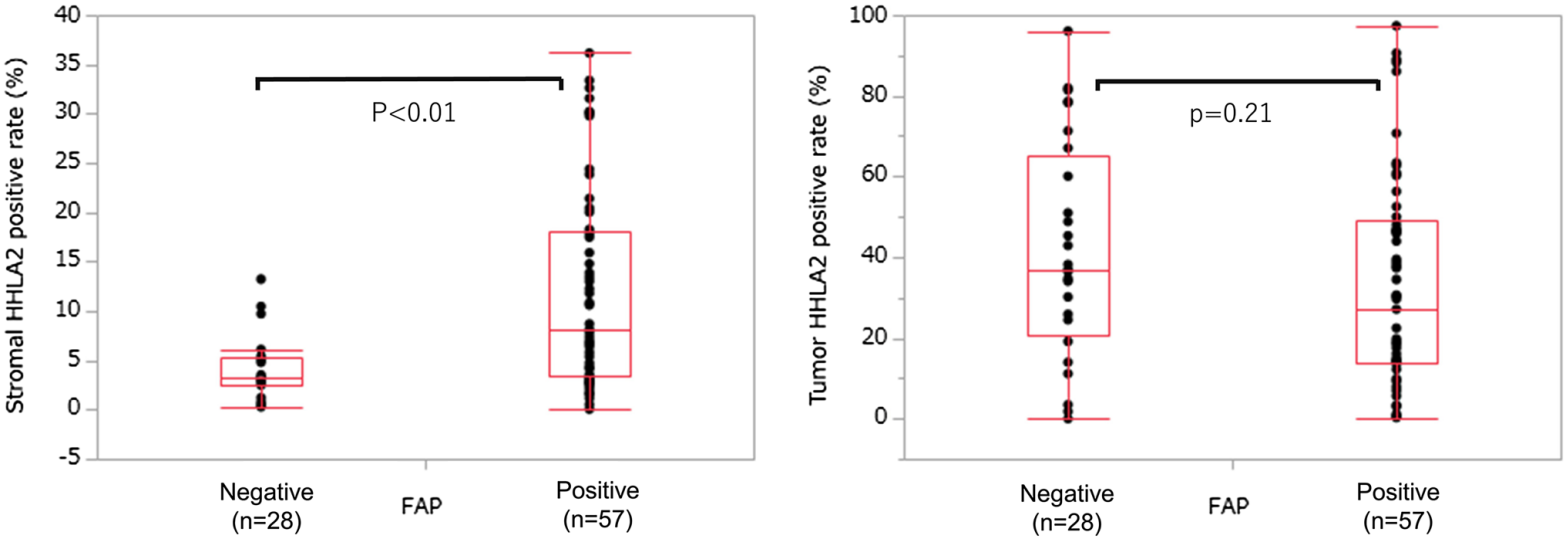
Relationship between the stromal FAP expression and stromal and tumor HHLA2 expression. Expression of stromal FAP is positively associated with that of stromal HHLA2 but not tumor HHLA2

Immunostaining with an anti-FAP antibody detected the expression of FAP in stromal cells, which are assumed to be CAFs, but not in tumor cells (Fig. 1E, F). A positive correlation was observed between the expressions of s-HHLA2 and s-FAP (*p* < 0.01, Fig. 3A). However, no correlation was observed between the expressions of t-HHLA2 and s-FAP (*p* = 0.21, Fig. 3B). A positive s-FAP expression was related to a higher histological grade, higher stage, positive LVI, and higher C-reactive protein (Table 1). Although s-FAP expression was not significantly associated with CSS (Fig. 2E), PFS was significantly shorter in s-FAP positive patients than in s-FAP negative patients (Fig. 2F).

According to the univariate analysis, a higher histological grade, higher pathological T (pT) stage, positive LVI, higher NLR, and lower t-HHLA2 expression were associated with worse CSS. Based on a stepwise multivariate analysis, lower t-HHLA2 along with higher grade and higher pT stage were the predictors of shorter CSS (Table 2). In contrast, based on the univariate analysis, a higher grade, higher pT stage, positive LVI, lower t-HHLA2 expression, and positive s-FAP expression were associated with a shorter PFS. Stepwise multivariate Cox’s proportional hazards analysis revealed that higher pT stage and positive LVI were significant predictors of shorter PFS (Table 3).

**Table 2:** Cox's proportional hazard analysis for cancer-specific survival

			Univariate analysis			Multivariate analysis (full model)			Multivariate analysis (reduced model)		
Variables	Category	*N*	HR	95% CI	*P* value	HR	95% CI	*P* value	HR	95% CI	*P* value
t-HHLA2	High/low (ref.)	58/27	0.26	0.10–0.61	< 0.01*	0.41	0.15–1.1	0.06	0.41	0.16–0.97	0.04*
s-HHLA2	High/ low (ref.)	47/38	1.9	0.79–4.9	0.16	1.7	0.64–4.8	0.31			
FAP	Positive/negative (ref.)	57/28	2. 5	0.93–8.6	0.07	0.51	0.13–2.3	0.36			
Grade	3 / ≤ 2 (ref.)	34/51	7.0	2.8–21	< 0.01*	2.3	0.69–8.9	0.18	3.51	1.26–11.5	0.01*
T stage	≥ 3 / ≤ 2 (ref.)	37/48	7.8	2.9–27	< 0.01*	5.2	1.4–23	0.01*	4.26	1.45–15.7	< 0.01*
LVI	1 / 0 (ref.)	34/51	7.2	2.8–22	< 0.01*	2.2	0.64–8.5	0.21			
NLR	> 3.500 / ≤ 3.500 (ref.)	12/73	2.9	1.0–7.1	0.04*	0.65	0.19–1.9	0.44			
TIL in stromal cells	> 5% / ≤ 5% (ref.)	73/12	4.3	0.90–78	0.07	1.6	0.31–29	0.63			

*CI* confidence interval, *FAP* fibroblast activation protein, *HR* hazard ratio, *LVI* lymphovascular invasion, *NLR* neutrophil lymphocyte ratio, *TIL* tumor-infiltrating lymphocyte

An asterisk (*) indicates a statistical significance (*p*-value < 0.05)

**Table 3:** Cox's proportional hazard analysis for progression-free survival

			Univariate analysis			Multivariate analysis (full model)			Multivariate analysis (reduced model)		
Variables	Category	*N*	HR	95% CI	*P* value	HR	95% CI	*P* value	HR	95% CI	*P* value
t-HHLA2	High/low (ref.)	58/27	0.46	0.23–0.93	0.03*	0.74	0.34–1.6	0.45			
s-HHLA2	High/low (ref.)	47/38	1.4	0.68–2.8	0.39	1.0	0.47–2.2	0.98			
FAP	Positive/negative (ref.)	57/28	2.7	1.2–7.3	0.02*	0.58	0.20–1.9	0.34			
Grade	3 / ≤ 2 (ref.)	34/51	4.2	2.1–8.8	< 0.01*	1.2	0.52–2.9	0.66			
T stage	≥ 3 / ≤ 2 (ref.)	37/48	9.0	4.0–24	< 0.01*	6.0	2.1–20	< 0.01*	5.18	2.15–14.6	< 0.01*
LVI	1 / 0 (ref.)	34/51	7.8	3.6–18	< 0.01*	4.2	1.6–12	< 0.01*	4.30	1.90–10.7	< 0.01*
NLR	> 3.500 / ≤ 3.500 (ref.)	12/73	2.4	1.0–5.1	0.04*	0.63	0.24–1.5	0.32			
TIL in stromal cells	> 5% / ≤ 5% (ref.)	73/12	7.0	1.5–125	< 0.01*	3.9	0.81–70	0.10			

*CI* confidence interval, *FAP* fibroblast activation protein, *HR* hazard ratio, *LVI* lymphovascular invasion, *NLR* neutrophil lymphocyte ratio, *TIL* tumor-infiltrating lymphocyte

An asterisk (*) indicates a statistical significance (*p*-value < 0.05)

## Discussion

In this study, we observed that the elevated expression of t-HHLA2 was related to a lower histological grade of cancer and longer CSS and PFS. Moreover, an increased expression of s-HHLA2 was associated with a higher histological grade but not with survival. Lastly, a positive association was observed between the expressions of s-HHLA2 and s-FAP, and an increased s-FAP expression correlated with a higher histological grade, higher pT stage, and shorter PFS. These findings suggest that the expressions of HHLA2 and FAP in the TME influence UTUC progression.

The detailed mechanism elucidating the effect of HHLA2 expression in the TME on tumor progression is yet to be established. In gastric cancer, lower levels of HHLA2 mRNA in the blood was reportedly associated with tumor aggressiveness, adverse prognosis, and lesser 5-year survival rates [16], whereas the overexpression of HHLA2 in cancer tissues was correlated with poor overall survival [17]. In this study, an increased t-HHLA2 expression was associated with negative LVI, lower NLR, and better survival, whereas an increased s-HHLA2 expression was associated with a higher histological grade and shorter survival. These observations indicate that HHLA2 plays a co-stimulatory role in tumor cells and a co-inhibitory role in stromal cells in the TME. Some studies have reported an interaction between tumor cells, T cells, and stromal cells to regulate HHLA2 expression [18, 19]. Thus, clarifying the mechanism of HHLA2 expression in the tumor and stromal cells in the TME may lead to new therapeutic approaches. The T cell receptor recognition of the cognate antigen presented by major histocompatibility complex molecules on the surface of cancer cells results in T cell activation [20]. Therefore, the relationship between the expressions of CD4/CD8 and HHLA-2 should also be studied. If s-HHLA2 expression is positively correlated to the intratumoral infiltration of CD8-positive T cells, it indicates that HHLA-2 expression is induced by immune responses by CD8-positive T cells.

Our findings suggest that t-HHLA2 and s-HHLA2 may produce different effects on the progression of UTUC, warranting further research on the effects of the stromal cells in the tumor environment. The TME is comprised of the mesenchymal stromal cells, activated fibroblasts, immune cells, capillaries, basement membrane, and the extracellular matrix surrounding the tumor cells, and mediates cancer progression and metastasis [21]. Several cell types may transit to the tumor and differentiate into CAFs. The CAFs in the TME support the growth and invasion of epithelial cells by secreting cytokines, chemokines, and epithelial–mesenchymal transition components that promote epithelial–mesenchymal transition. Moreover, CAFs express FAP which facilitates epithelial–mesenchymal transition. FAP expressed by CAFs is highly upregulated in various cancers and is typically used as a prognostic marker [12]. Although the detailed mechanism is yet to be elucidated, multiple environmental and soluble factors are known to alter FAP expression.

An elevated FAP expression in CAFs was associated with a higher stage and poor disease-specific survival in bladder urothelial cancer [22]. In this study, the increased expression of s-FAP correlated with a higher pT stage and poor prognosis, indicating that s-FAP is associated with biological aggressiveness and UTUC progression. Although a positive relationship was observed between the expressions of s-HHLA2 and s-FAP, it is currently unclear whether HHLA2 and FAP act cooperatively or independently in stromal tissues. Future studies could also examine the relationship between HHLA2 and CAFs in the stromal microenvironment, and whether CAFs induce tumor progression by modulating the immunosuppressive stromal microenvironment through the activation of s-HHLA2.

Research on the TME has yielded increasing evidence that a greater systemic inflammatory response is associated with poor outcomes. The NLR, which is related to the systemic inflammatory response, may predict solid tumors, including UTUC, because a high NLR is associated with poor survival [23]. The mechanism through which a higher NLR leads to a poor prognosis is unclear; however, local immunosuppression mediated by cytokines has been proposed as a possible mechanism. The inflammatory response activates neutrophils, which act as immunosuppressants by decreasing the activity of lymphocytes, activated T cells, natural killer cells, and other immune cells [24, 25]. This study revealed that increased t-HHLA2 expression is related to lower NLR, and increased s-FAP expression is related to higher CRP levels, which is also a cancer biomarker. However, such associations were not observed for s-HHLA2, suggesting that HHLA2 and CAFs may be associated with the systemic inflammatory response in the UTUC microenvironment. Moreover, inflammation is known to be involved in the progression of tumors [24, 25]. Hence, the connection between HHLA2/FAP expression and other serum inflammatory markers, like the systemic immune-inflammation and systemic inflammation response indices, should also be studied.

The limitations of this study include the relatively small sample size and short follow-up. The small sample size may be responsible for Cox’s proportional hazard analysis not detecting significant associations between HHLA2/FAP expressions and survival outcomes. Although HHLA2 and FAP are expected to be associated with anti-tumor immune response, we could not verify whether the expression of HHLA2 and FAP affects the efficacy of immune checkpoint inhibitors, because only a few cases used immune checkpoint inhibitors in this study. Furthermore, this study did not explore whether HHLA2 and FAP jointly or separately increase or decrease the anti-tumor immune response or the mechanism involved, and this should be investigated further. Thus, to elucidate the molecular mechanism of immune regulation by HHLA2, we should study the HHLA2/FAP receptors in the tumor region and stromal tissues through colocalization experiments and the use of multiple immunostaining analyses using a panel of proteins, such as CAFs and immune-regulated proteins, including HHLA2, TMIGD2, and KIR3DL3, in the TME. As studies have reported that tumor-infiltrating lymphocytes may be associated with survival [26, 27], the association between tumor-infiltrating lymphocytes and HHLA2 expression should be evaluated, and the percentage of tumor-infiltrating lymphocytes and the outcomes should be analyzed. The mechanism of signal transduction between HHLA2 and FAP in the TME should also be determined, elucidating the mechanism through which HHLA2 suppresses and stimulates the immune response.

## Data Availability

The datasets generated and analysed during the current study are available from the corresponding author on reasonable request.
